# Skin Commensal Bacteria Modulates the Immune Balance of Mice to Alleviate Atopic Dermatitis-Induced Damage

**DOI:** 10.1155/2022/4731675

**Published:** 2022-09-17

**Authors:** Xianshui Yu, Ting Chen, Ning Huang, Yanxia Jin, Ling Yang

**Affiliations:** ^1^Department of Dermatology, 920th Hospital of Joint Logistics Support Force of Chinese People's Liberation Army, Kunming, Yunnan 650032, China; ^2^Department of Pharmacy, 920th Hospital of Joint Logistics Support Force of Chinese People's Liberation Army, Kunming, Yunnan 650032, China

## Abstract

**Objective:**

Although studies indicate that *Staphylococcus epidermidis* (*S. epidermidis*) can regulate inflammation and anti-inflammatory cytokines, there is limited evidence supporting their effects on atopic dermatitis (AD). Here, we aimed to investigate the effects and potential mechanism of skin commensal bacteria on the immunity of mice with AD.

**Methods:**

Twenty-four female BALB/C mice were selected and divided randomly into 4 groups: normal group, atopic dermatitis model group (AD), atopic dermatitis/substrate group (AD/substrates), and atopic dermatitis/substrates/epidermidis group (AD/*S. epidermidis*). All the mice were given different ways. After 14 days, their skin conditions were scored, and the serum, ear tissue, and inguinal lymph node tissue were collected and analyzed. Furthermore, the flow cytometry was used to analyze the number of CD4°+°CD25°+°Foxp3°+°Treg in the mouse lymph node tissue.

**Results:**

Compared with the AD/substrate group, the mice ear thickness and dermatitis score were significantly reduced in the AD/*S. epidermidis* group; skin epidermis, acanthosis, the degree of keratinization, inflammatory cell infiltration in the dermis, and the number of mast cells were declined. The serum levels of IgE, IgG1, IgG2a, and TNF-*α*, IFN-*γ*, IL-4, and Eotaxin were significantly declined in the AD/*S. epidermidis* compared with the AD/substrate group. The proportion of CD4°+°CD25°+°Foxp3°+°Treg cells in the lymph node tissue was significantly increased in the AD/*S. epidermidis* group compared with the AD/substrate group.

**Conclusion:**

*Staphylococcus epidermidis* can regulate mice's immune balance to alleviate AD-induced skin damage.

## 1. Introduction

Atopic dermatitis (AD) is one of the most common chronic inflammatory skin diseases that mainly affects children [1], with a 20% prevalence in infants under two years old and 3% in adults worldwide [2]. Its prevalence has increased by 2 to 3-fold during the past decades in industrialized countries [3]. In America, it was found that the incidence of AD was approximately 10.7% (2011) in children and 7.2% (2015) in adults [4]. The main symptoms of AD are the destruction of the stratum corneum, eczema, and pruritus. Pruritus-caused skin scratches can severely damage the skin epidermal barrier, and subsequently, immunomodulatory proteins such as tumor necrosis factor (TNF)-*α*, interferon (IFN)-*γ*, and interleukin (IL-4) accumulate in the skin epithelial cells [5]. Immunomodulatory proteins are known to initiate and promote T helper (Th) 2 cell-mediated immune responses and stimulate B cells to undergo isotype switching from immunoglobulin (Ig) M to E [6]. However, increased IgE can result in the accumulation of eosinophil granulocytes in the dermis [7], thereby increasing immune imbalance-caused skin inflammation and aggravating AD symptoms. Besides, patients with AD are at increased risk of other atopic diseases, including asthma, allergic rhinitis, and food allergy [8]. Currently, AD treatment is focused on improving the severity of skin inflammation, including treatment with topical corticosteroids and calcineurin inhibitors, ultraviolet light, and systemic immunosuppression [9]. However, drugs such as corticosteroids, calcineurin inhibitors (i.e., cyclosporine), and JAK inhibitors can cause side effects such as nausea, vomiting, diarrhea, skin thinning, and purpura [10]. In addition, the FDA has added a boxed warning to topical calcineurin inhibitors. Phototherapy is an alternative treatment option, but its use is limited due to high cost, inadequate local availability, and poor patient compliance [11]. Therefore, there is a need to develop alternative AD treatments.

The human skin is a complex barrier organ colonized by beneficial microorganisms interacting with their surroundings, including the host and other microbes. These interactions boost the barrier function of the skin. Therefore, a good symbiotic relationship between microbial communities is essential for the healthy skin [12]. Skin dysbiosis, defined as an imbalance of skin microbial organisms [13], is associated with an altered immune response and promotes the development of skin diseases. There is ample evidence showing that the skin of AD patients is more prone to *Staphylococcus aureus (S*. *aureus)* colonization and overgrowth [14]. Therefore, maintaining or restoring the skin microbial structure while specifically eliminating *S*. *aureus* is an ideal treatment strategy for AD.

*Staphylococcus epidermidis* belongs to a class of coagulase-negative staphylococci (CNS) and is a harmless commensal bacterium abundant on the human skin [15]. In natural ecology, *Staphylococcus epidermidis* plays a very important role in maintaining local homeostasis. In addition, it may compete with other potentially harmful microorganisms, such as *S*. *aureus*, to maintain a healthy skin flora. It is reported that polysaccharide adhesins on the surface of *Staphylococcus epidermidis* can activate human astrocytes through toll-like receptors (TLR) [16], and peptidoglycan (PG) derived from the astrocytes can activate human mononuclear THP-1 cells [17]. Some studies have reported that *Staphylococcus epidermidis* can also regulate the occurrence of IFN-*γ*, L-18, IL-12, and TNF [18]. According to the related reports, *Staphylococcus epidermidis* has a specific immunoregulatory function and can regulate the occurrence of inflammation and anti-inflammatory cytokines [19, 20]. The activation of immunoregulatory functions and regulation of cytokine occurrence are important defense mechanisms to prevent further damage by AD.

However, there are presently few studies on the effects of *Staphylococcus epidermidis* on AD. Therefore, in this study, an AD mouse model was established to explore the effects of *Staphylococcus epidermidis* on AD, with the objective of offering new methods and data for the clinical treatment of AD.

## 2. Materials and Methods

### 2.1. Experimental Animal

Twenty-four female SPF BALB/C mice (age: eight weeks) were housed in a temperature-controlled environment (202-21°C) with a relative humidity of 40–45% and 12 h light/dark cycles. After one week of standard diet feeding, the mice were taken through a follow-up test. This study was approved by the Ethics Committee of the 920th Hospital of Joint Logistics Support Force of Chinese People's Liberation Army (no. 2022-01-01), and the experiment was performed according to relevant guidelines and regulations.

### 2.2. Establishment of the Atopic Dermatitis Mouse Model

The female BALB/C mice were randomly divided into four groups (six mice per group). Mice without any treatment were set as the normal group. In the AD group, the mice's ears were painted with 2 nmol of MC903, a low-calcemic vitamin D3 analog, for 10 consecutive days [21, 22]. The topical application of MC903 induces the thymic stromal lymphopoietin (TSLP) expression in epidermal keratinocytes resulting in skin morphology changes and inflammation similar to immune perturbations observed in acute lesions of AD patients [22]. After one week of standard diet feeding, the hair on the back of the mice was removed. One fingertip unit of emulsion matrix was applied on the back of the mice twice a day (once in the morning and once in the afternoon). The mice were treated differently. For the AD/substrate group, the mice were painted with one fingertip unit of emulsion matrix on the back for seven successive days and with 2 nmol of MC903 on the ears. In the AD/*S. epidermidis* group, from the third day of painting with the emulsion matrix, the mice were painted with 150 *μ*L of *Staphylococcus epidermidis* (10^7^ CFU/mL; ATCC, USA) on the back once a day. After painting for five successive days, the ears were painted with 2 nmol of MC903. After 14 days, the serum, ear tissue, and inguinal lymph node tissue of the mice were collected.

### 2.3. Skin Severity Score

The mice ear thickness in each group was measured daily at the start of stimulation with MC903. Their skin conditions were scored (signs and symptoms: erythema/hemorrhage, edema, excoriation/erosion, scaling/dryness, and itch), and the scores were graded as 0 (none), 1 (mild), 2 (moderate), and 3 (severe) based on severity [23].

### 2.4. H and E Staining

Each mouse's right ear tissue was fixed with 4% paraformaldehyde for 48 h. Subsequently, the tissue was embedded and cut into 4-*μ*m-thick sections. After deparaffinization with xylene, the sections were stained with hematoxylin for 5 min at an ambient temperature. Then, hydrochloric acid in ethanol was applied for tissue differentiation. After washing with water, the tissue was stained with eosin for 30 s. Then, alcohol was used to dehydrate. After treatment with dimethylbenzene, the tissue was mounted. The degree of keratinization and epidermal hyperplasia in the mouse tissue in each group were observed under a biomicroscope, and the thickness of the skin tissue *epidermis* was quantitatively analyzed.

### 2.5. Toluidine Blue Staining

After deparaffinization and hydration, sections of mice's right ear tissue were stained with 1% toluidine blue for approximately 20 minutes. Then, the sections were slowly rinsed with water for about 30 seconds, followed by using 95% alcohol for color separation. The sections were cleared with xylene for 2 min and finally mounted with neutral gum. The mast cell infiltration was observed under a biomicroscope, and the thickness of the skin tissue *epidermis* was quantitatively analyzed.

### 2.6. ELISA Detection

The mice's whole blood was centrifuged at 3000 r/min at 4°C for 20 min, and the supernatant was collected. Their ear tissue was placed in a 2 mL tube for homogenizing. Homogenization beads and precooled PBS buffer (1 mL) were added to the tube. Then, the tissue was homogenized for 3 min at 50 Hz. After centrifugation at 12000 r/min at 4°C for 20 min, the supernatant was collected. The level of IgE, IgG1, and IgG2a in the serum and TNF-*α*, IFN-*γ*, IL-4, and Eotaxin in the mouse ear tissue in each group was evaluated following the ELISA kit instructions (MultiSciences (Lianke) Biotech Co., Ltd., China).

### 2.7. qRT-PCR

TRizol reagent (ThermoFisher, America) was used to extract the total RNA in the mouse ear tissue. Then, NanoDrop was used to check the concentration and purity of RNA. The cDNA was prepared following the random primer reverse transcription kit (Takala, Japan). The expression of TNF-*α*, IFN-i, IL-4s, and Eotaxin's mRNA was determined according to the instructions of the SYBR GREEN kit. GAPDH was used as an internal control for the analysis of the trial. The experiment was set up for 6 replicates. The 2^−ΔΔCt^ method was applied to calculate the relative expression of target genes according to the data obtained from qRT-PCR. Primer sequences are shown in Supplementary Table 1.

### 2.8. CD4°+°CD25°+°Foxp3°+°Treg Cell Assay

Surface fat and fibers of the lymph node tissue blocks of 5 mm^3^ size were removed. After PBS was added to a 100-mesh stainless-steel mesh, the tissue was ground into cell suspension, which was then filtered using a 200-mesh nylon screening. After cell aggregates and uncrushed cell blocks were removed, the suspension was centrifuged. Then, PBS was used to resuspend for the preparation of single-cell suspension. Human lymphocytes separating medium was added to obtain mononuclear cells. After adding 2.5 *μ*L of Percp-Cy5.5-CD4 monoclonal antibody and PE-CD25 monoclonal antibodies, the cells were incubated at 4°C in the dark for 30 min. Subsequently, permeabilization wash buffer was added. Then, 2.5 *μ*L of APC-Foxp3 antibody was added, and the cells were incubated at 4°C for 30 min. Flow cytometry and the Cellquest software were used to evaluate and analyze the cells, respectively, and the ratio of Tregs cells in CD4 + cells was recorded.

### 2.9. Statistical Analysis

The results were expressed as mean ± standard deviation (SD). *T*-test analysis was utilized for the two-group comparisons and a one-way analysis for multigroup comparisons. All data were analyzed using the SPSS 25.0 software. Differences were considered to be statistically significant when *P* < 0.05.

## 3. Results

### 3.1. Skin Commensal Bacteria Can Alleviate MC903-Induced Atopic Dermatitis Symptoms in Mice

**Figure 1:**
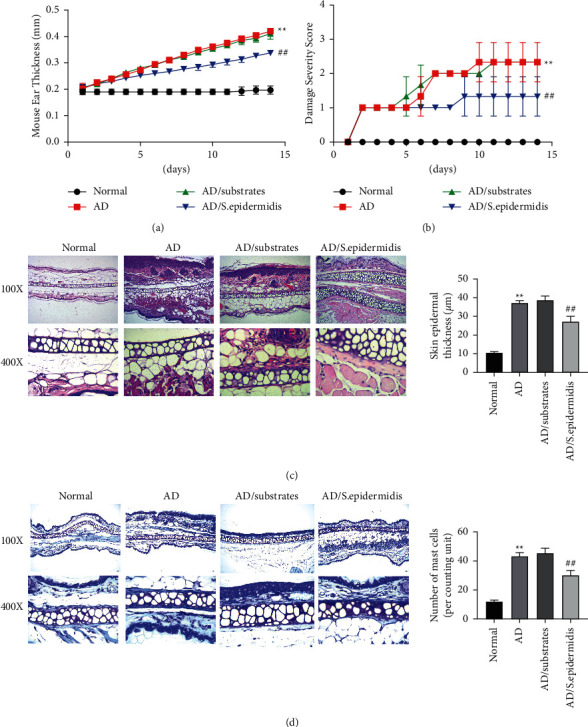
Effects of skin commensal bacteria on MC903-induced atopic dermatitis symptoms in mice. (a) The ear thickness of the mice in each group; (b) dermatitis severity score of mice in each group; (c) H and E staining was applied to observe the mouse ear tissue structure; (d) toluidine blue was used to observe infiltration of mast cells in the mouse ear tissue. ^##^*P*  <  0.01*vs*. normal group and ^##^*P*  <  0.01*vs*. AD/substrate group.

The effect of skin commensal bacteria on AD symptoms in mice was first evaluated. The results revealed that, compared with the normal group, the ear thickness and dermatitis severity score of the mice in the AD and AD/substrate groups were significantly increased. Notably, after painting with skin commensal bacteria, both the ear thickness and severity score were significantly reduced in the AD/*S. epidermidis* group compared with the AD/substrate group (Figures 1(a) and 1(b)).

The effects of skin commensal bacteria on the skin structure and the number of mast cells were evaluated. The results indicated that the skin tissue structure in the normal group was intact and normal, and there were no obvious abnormal changes in the dermis. In the AD and AD/substrate groups, the skin structure was severely damaged, with thickened *epidermis*, hyperkeratosis, acanthosis, edema of epidermal cells, dermal vasodilatation, and massive inflammatory cell infiltration. However, in the AD/*S. epidermidis* group, thickened *epidermis* and acanthosis were alleviated, the degree of keratinization was reduced, and dermal inflammatory cell infiltration was decreased compared with the AD/substrate group (Figure 1(c)). According to the toluidine blue staining results, compared with the normal group, the number of mast cells was significantly increased in the AD and AD/substrate groups. Notably, the number of mast cells in the AD/*S. epidermidis* group was reduced compared with the AD/substrate group (Figure 1(d)). Together, these results suggested that skin commensal bacteria could alleviate skin damage and reduce AD severity in mice.

### 3.2. Skin Commensal Bacteria Can Restore Immune Balance and Decrease the Inflammatory Factor Level in Mice with Atopic Dermatitis

**Figure 2:**
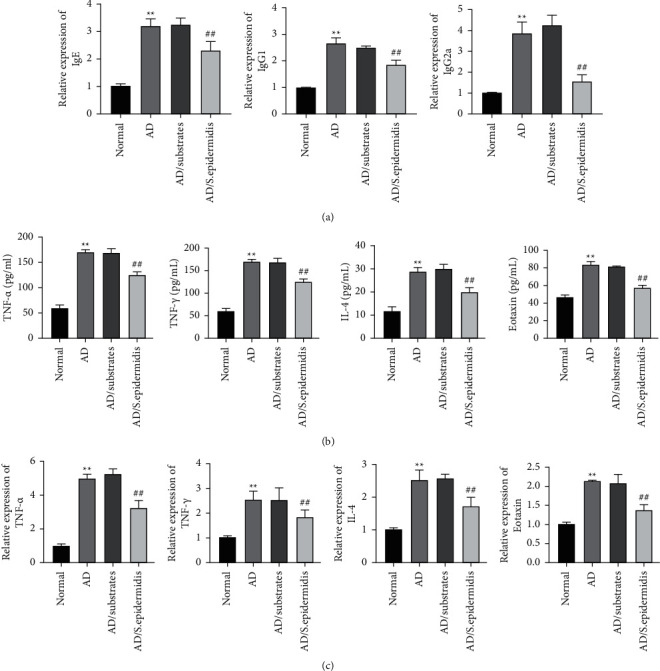
Effects of skin commensal bacteria on immune and inflammatory factors of mice with atopic dermatitis. a andb, ELISA was used to check the level of IgE, IgG1, and IgG2a in the mice serum (a); and TNF-*α*, IFN-*γ*, IL-4, and Eotaxin in the mouse ear tissue (b); and (c) QRT-PCR was used to measure the expression of TNF-*α*, IFN-*γ*, IL-4, and Eotaxin mRNA in the mouse ear tissue. ^*∗∗*^*P* < 0.01*vs*. normal group and ^##^*P*  <  0.01*vs*. AD/substrate group.

The effects of skin commensal bacteria on immune and inflammatory factors were evaluated. Compared with the normal group, we found that the levels of immune factors (IgE, IgG1, and IgG2a), inflammatory factors (TNF-*α*, IFN-*γ*, and IL-4), and the expression and release levels of mRNA in Eotaxin were significantly upregulated in the serum of the AD and AD/substrate mice groups. However, there were no significant differences in these factors between the AD and AD/substrate groups. Notably, after skin commensal bacteria were added, both the immune and inflammatory factors in the serum were decreased in the AD/*S. epidermidis* compared with the AD/substrate group (Figures 2(a)–2(c)). Together, these results indicated that skin commensal bacteria might affect the immune balance and inflammatory reaction, relieving AD symptoms in mice.

### 3.3. Skin Commensal Bacteria Can Restore the Proliferation of CD4°+°CD25°+°Foxp3°+°Treg Cell in the Lymph Nodes

**Figure 3:**
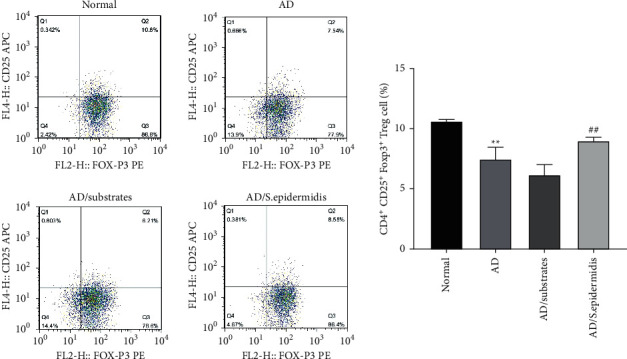
Effects of skin commensal bacteria on the proportion of CD4+ CD25+ Foxp3+Treg cells in the lymph node tissue of mice with atopic dermatitis. ^*∗∗*^*P* < 0.01 vs. normal group and ^##^*P*  <  0.01 vs. AD/substrate group.

Flow cytometry was used to determine the effects of skin commensal bacteria on the recovery of immune function in AD mice. The results revealed that the proportion of CD4°+°CD25°+°Foxp3°+°Treg cells in the mice lymph node tissue in the AD and AD/substrate groups was significantly lower than that in the normal group. The proportion of CD4°+°VCD25°+°Foxp3°+°Treg cells in the lymph node tissue was significantly increased in the AD/*S. epidermidis* group compared with the AD/substrate group (Figure 3). These results indicated that skin commensal bacteria could improve the immune function of AD mice.

## 4. Discussion

Atopic dermatitis is a refractory skin disease characterized by recurrent and difficult-to-recover eczema and pruritus [24]. While its prevalence has been increasing worldwide, the etiology of AD is not yet well understood, and there is currently no effective AD treatment. The current AD standard medical therapies, including topical corticosteroids and topical calcineurin inhibitors [24], can improve the condition of AD patients but have a lot of side effects. *S. aureus* skin colonization is considered an important factor in the pathogenesis of AD [25]. It was shown to exacerbate skin inflammation and allergic reactions by disrupting the adaptive and innate immune responses through several mechanisms [26]. On the other hand, *Staphylococcus epidermidis* is one of the most abundant species in the skin microbiota and has been shown to limit the growth of *Staphylococcus aureus* [27]. Based on these findings, we hypothesized that restoring immune balance through *Staphylococcus epidermidis* would be an effective AD treatment strategy. Expectedly, this study showed that *Staphylococcus epidermidis* significantly reduced dermatitis severity and skin damage in MC903-induced AD mice.

In a recent meta‐analysis by Totte et al. [28], the authors reported that *Staphylococcus aureus* was significantly more prevalent on the lesional skin of AD patients than on the nonlesional skin of the same patients or the skin of healthy controls (70% vs. 39%), and its rate of colonization was shown to associate with disease severity. Although *S. epidermidis* is considered a beneficial skin microbe against *Staphylococcus aureus*, a recent study by Cau et al. [29] found that an overabundance of *Staphylococcus epidermidis* on some patients had similar harmful effects as *Staphylococcus aureus*. It was shown that some *Staphylococcus epidermidis* strains could impair the skin barrier and promote inflammation through the secretion of the cysteine protease EcpA, thereby increasing the disease severity [29]. Thus, *Staphylococcus epidermidis* may shift from a beneficial commensal to a deleterious pathogen similar to *Staphylococcus aureus* in the permissive growth conditions of the AD skin. Some unique coagulase‐negative staphylococci (CoNS) strains, such as *Staphylococcus hominis A9* and *C5*, were shown effective in inhibiting and preventing *S. epidermidis*‐induced skin damage [29]. *S. hominis A9* was shown to kill *S. aureus*, whereas *Staphylococcus hominis C5* synthetic autoinducing peptide was shown to inhibit the *Staphylococcus aureus* [29]. These indicate that such commensal CoNS strains are promising therapeutic tools in reducing *Staphylococcus aureus* colonization and the toxin production of both *Staphylococcus aureus* and *Staphylococcus epidermidis* in AD. The above findings indicate the complexity of the interactions between commensal CoNS and deleterious *S. aureus* and suggest that using *Staphylococcus epidermidis* as the treatment for dysbiosis in AD may further damage the skin and cause inflammation. Thus, a small molecule with a narrow‐spectrum effect, especially on *Staphylococcus aureus*, might be an attractive alternative for the treatment of AD [30].

Mast cells are hematopoietic cells originating from bone marrow progenitor cells [31]. Mast cell-committed progenitors migrate through the circulation into the destination tissues and proliferate and differentiate into mature mast cells under the influence of the local microenvironment [32]. Mast cells are the key effector cells in allergic reactions. They are activated by IgE receptors, leading to degranulation and subsequent release of various inflammatory mediators such as histamine, serotonin, and tumor necrosis factor-*α* (TNF-*α*) [33, 34]. Most studies have shown an increase in the number of mast cells in skin lesions in AD models, so it is generally believed that mast cells contribute to skin inflammation [35]. The results of this study showed that *Staphylococcus epidermidis* decreased mast cell infiltration in mice ear tissues. These findings are consistent with those of Meng et al. who found that paeonol inhibited the development of 1-chloro-2,4-dinitrobenzene-induced AD in mice by differentiating mast cells and *T* cells [36].

AD is characterized by inflamed skin, impaired skin barrier function, and IgE-mediated sensitization to food and environmental allergens [37]. Currently, there are two main hypotheses explaining the pathogenesis of AD: (1) immune dysregulation causes Th2-predominant inflammation and IgE-mediated sensitization [38]; (2) intrinsic defects in skin barrier function. In addition, various inflammatory cytokines, mainly Th2 cell-derived cytokines, regulate and guide the nature of AD, including IL-4, TNF-*α*, IFN-*γ*, and Eotaxin [39]. IL-4 and IL-13 are the key drivers of the IgE isotype class switching, inflammation, and expression of receptors on the surface of mast cells. IL-4 and IL-13 often activate the IL-4 receptor (IL-4R) to downregulate skin barrier proteins, thus affecting the skin barrier [40]. Therefore, reducing the expression of IL-4 and IL-13 is an effective AD treatment strategy. The results of this study showed that *Staphylococcus epidermidis* could reduce the level of TNF-*α*, IFN-*γ*, IL-4, and Eotaxin in the ear tissue and the level of IgE, IgG1, and IgG2a in mice's serum.

Numerous studies have shown that clinical symptoms of AD patients can be relieved by modulating the immune system. Simon et al. found that immunosuppressive drugs (i.e., cyclosporine and a mycophenolate mofetil) significantly improved the AD symptoms [41]. Beck et al. found that the symptoms of AD could be significantly alleviated by anti-IL-4 receptor antibody or anti-B cell antibody [42]. These findings suggested that immune dysfunction exerted a crucial role in the pathogenesis of AD. CD4°+°regulatory T cells (Treg) are indispensable in maintaining immune self-tolerance and homeostasis in the normal immune system, accounting for 10% of all the CD4°+°T cells in the peripheral circulation [43, 44]. Treg cells have been shown to prevent excessive inflammation and maintain immune tolerance. However, dysfunctional Treg can lead to autoimmune diseases, including AD, systemic lupus erythematosus, and asthma [45]. Based on the results of this study, we found that *Staphylococcus epidermidis* could significantly increase the proportion of CD4°+°CD25°+°Foxp3°+°Treg cells in the lymph nodes of AD mice. These findings suggested that *Staphylococcus epidermidis* could regulate mice's immune balance to reduce AD-induced damage.

## 5. Conclusion

*Staphylococcus epidermidis* alleviated MC903-induced skin damage and improved AD symptoms in mice with AD. It may exert its function by reducing immune proteins, alleviating inflammation, inducing the proliferation of CD4°+°CD25°+°Foxp3°+°Treg cells, and restoring the immune function of AD mice. However, the target of *Staphylococcus epidermidis* in exerting its biological function remains unknown. Therefore, further studies are needed to comprehensively elucidate the specific mechanism of *Staphylococcus epidermidis* in regulating immune balance, thereby providing more accurate theory evidence for clinical application.

## Data Availability

The data used to support the findings of this study are available from the corresponding author upon request.

## Supplementary Materials

Supplementary Materials Supplementary Table 1: primer sequences used in qRT-PCR.

## References

[1] Zheng T., Yu J., Oh M. H., Zhu Z. (2011). The atopic march: progression from atopic dermatitis to allergic rhinitis and asthma. *Allergy, Asthma & Immunology Research*.

[2] Boguniewicz M., Leung D. Y. (2010). Recent insights into atopic dermatitis and implications for management of infectious complications. *The Journal of Allergy and Clinical Immunology*.

[3] Nutten S. (2015). Atopic dermatitis: global epidemiology and risk factors. *Annals of Nutrition & Metabolism*.

[4] Drucker A. M., Wang A. R., Li W. Q., Sevetson E., Block J. K., Qureshi A. A. (2017). The burden of atopic dermatitis: summary of a report for the national eczema association. *Journal of Investigative Dermatology*.

[5] Nygaard U., Hvid M., Johansen C. (2016). TSLP, IL-31, IL-33 and sST2 are new biomarkers in endophenotypic profiling of adult and childhood atopic dermatitis. *Journal of the European Academy of Dermatology and Venereology*.

[6] Misery L. (2014). La TSLP, clé du prurit dans la dermatite atopique. *Medical Science*.

[7] Hellman L. T., Akula S., Thorpe M., Fu Z. (2017). Tracing the origins of IgE, mast cells, and allergies by studies of wild animals. *Frontiers in Immunology*.

[8] Long H., Zhang G., Wang L., Lu Q. (2016). Eosinophilic skin diseases: a comprehensive review. *Clinical Reviews in Allergy and Immunology*.

[9] Cabanillas B., Brehler A. C., Novak N. (2017). Atopic dermatitis phenotypes and the need for personalized medicine. *Current Opinion in Allergy and Clinical Immunology*.

[10] Klonowska J., Glen J., Nowicki R. J., Trzeciak M. (2018). New cytokines in the pathogenesis of atopic dermatitis-new therapeutic targets. *International Journal of Molecular Sciences*.

[11] Patrizi A., Raone B., Ravaioli G. M. (2015). Management of atopic dermatitis: safety and efficacy of phototherapy. *Clinical, Cosmetic and Investigational Dermatology*.

[12] Grice E. A., Segre J. A. (2011). The skin microbiome. *Nature Reviews Microbiology*.

[13] Kaur N., Chen C. C., Luther J., Kao J. Y. (2011). Intestinal dysbiosis in inflammatory bowel disease. *Gut Microbes*.

[14] Leung D. Y. (2013). New insights into atopic dermatitis: role of skin barrier and immune dysregulation. *Allergology International*.

[15] Stevens N. T., Sadovskaya I., Jabbouri S. (2009). Staphylococcus epidermidis polysaccharide intercellular adhesin induces IL-8 expression in human astrocytes via a mechanism involving TLR2. *Cellular Microbiology*.

[16] Gong L., Bao Q., Hu C. (2018). Exosomal miR-675 from metastatic osteosarcoma promotes cell migration and invasion by targeting CALN1. *Biochemical and Biophysical Research Communications*.

[17] Stuyt R. J., Kim S. H., Reznikov L. L. (2003). Regulation of staphylococcus epidermidis-induced IFN-gamma in whole human blood: the role of endogenous IL-18, IL-12, IL-1, and TNF. *Cytokine*.

[18] Bieber T. (2008). Atopic dermatitis. *New England Journal of Medicine*.

[19] Naik S., Bouladoux N., Linehan J. L. (2015). Commensal-dendritic-cell interaction specifies a unique protective skin immune signature. *Nature*.

[20] Nakatsuji T., Chen T. H., Narala S. (2017). Antimicrobials from human skin commensal bacteria protect against *Staphylococcus aureus* and are deficient in atopic dermatitis. *Science Translational Medicine*.

[21] Chang Z. F. Y. J., Zhang D., Wang L. Study on mechanism of compound glycyrrhizin in treatment of atopic dermatitis in mice based on miR-155/SOCS1 axis. *Chinese Journal of Immunology*.

[22] Li M., Hener P., Zhang Z., Kato S., Metzger D., Chambon P. (2006). Topical vitamin D3 and low-calcemic analogs induce thymic stromal lymphopoietin in mouse keratinocytes and trigger an atopic dermatitis. *Proceedings of the National Academy of Sciences of the United States of America*.

[23] Cox L., Calderon M. A. (2016). Allergen immunotherapy for atopic dermatitis: is there room for debate?. *Journal of Allergy and Clinical Immunology: In Practice*.

[24] Soumelis V. (2017). Molecular and cellular discoveries in inflammatory dermatoses. *Journal of the European Academy of Dermatology and Venereology*.

[25] Chiu L. S., Chow V. C. Y., Ling J. M. L., Hon K. L. (2010). *Staphylococcus aureus* carriage in the anterior nares of close contacts of patients with atopic dermatitis. *Archives of Dermatology*.

[26] Bekeredjian-Ding I., Inamura S., Giese T. (2007). *Staphylococcus aureus* protein A triggers T cell-independent B cell proliferation by sensitizing B cells for TLR2 ligands. *The Journal of Immunology*.

[27] Glatthardt T., Campos J. C. d. M., Chamon R. C. (2020). Small molecules produced by commensal Staphylococcus epidermidis disrupt formation of biofilms by *Staphylococcus aureus*. *Applied and Environmental Microbiology*.

[28] Totte J. E., van der Feltz W. T., Hennekam M., van Belkum A., van Zuuren E. J., Pasmans S. G. (2016). Prevalence and odds of *Staphylococcus aureus* carriage in atopic dermatitis: a systematic review and meta-analysis. *British Journal of Dermatology*.

[29] Cau L., Williams M. R., Butcher A. M. (2021). Staphylococcus epidermidis protease EcpA can be a deleterious component of the skin microbiome in atopic dermatitis. *The Journal of Allergy and Clinical Immunology*.

[30] Chu C. Y. (2022). Targeting the cutaneous microbiota in atopic dermatitis: ‘A new hope’ or ‘attack of the CoNS. *Clinical and Translational Medicine*.

[31] Kitamura Y., Ito A. (2005). Mast cell-committed progenitors. *Proceedings of the National Academy of Sciences of the United States of America*.

[32] Galli S. J., Maurer M., Lantz C. S. (1999). Mast cells as sentinels of innate immunity. *Current Opinion in Immunology*.

[33] Galli S. J., Tsai M. (2012). IgE and mast cells in allergic disease. *Nature Medicine*.

[34] Zhang B., Alysandratos K. D., Angelidou A. (2011). Human mast cell degranulation and preformed TNF secretion require mitochondrial translocation to exocytosis sites: relevance to atopic dermatitis. *The Journal of Allergy and Clinical Immunology*.

[35] Saluja R., Khan M., Church M. K., Maurer M. (2015). The role of IL-33 and mast cells in allergy and inflammation. *Clinical and Translational Allergy*.

[36] Meng Y., Liu Z., Zhai C. (2019). Paeonol inhibits the development of 1chloro2, 4dinitrobenzeneinduced atopic dermatitis via mast and T cells in BALB/c mice. *Molecular Medicine Reports*.

[37] Korosec P., Gibbs B. F., Rijavec M., Custovic A., Turner P. J. (2018). Important and specific role for basophils in acute allergic reactions. *Clinical and Experimental Allergy*.

[38] Eyerich K., Novak N. (2013). Immunology of atopic eczema: overcoming the Th1/Th2 paradigm. *Allergy*.

[39] Otsuka A., Nomura T., Rerknimitr P., Seidel J. A., Honda T., Kabashima K. (2017). The interplay between genetic and environmental factors in the pathogenesis of atopic dermatitis. *Immunological Reviews*.

[40] Lee D. E., Clark A. K., Tran K. A., Shi V. Y. (2018). New and emerging targeted systemic therapies: a new era for atopic dermatitis. *Journal of Dermatological Treatment*.

[41] Simon D., Hosli S., Kostylina G., Yawalkar N., Simon H. U. (2008). Anti-CD20 (rituximab) treatment improves atopic eczema. *The Journal of Allergy and Clinical Immunology*.

[42] Beck L. A., Thaci D., Hamilton J. D. (2014). Dupilumab treatment in adults with moderate-to-severe atopic dermatitis. *New England Journal of Medicine*.

[43] Sakaguchi S. (2004). Naturally arising CD4+ regulatory t cells for immunologic self-tolerance and negative control of immune responses. *Annual Review of Immunology*.

[44] Josefowicz S. Z., Lu L. F., Rudensky A. Y. (2012). Regulatory T cells: mechanisms of differentiation and function. *Annual Review of Immunology*.

[45] Sakaguchi S., Yamaguchi T., Nomura T., Ono M. (2008). Regulatory T cells and immune tolerance. *Cell*.

