# Supplementary material for: Skin Commensal Bacteria Modulates the Immune Balance of Mice to Alleviate Atopic Dermatitis-Induced Damage
Source: Evid Based Complement Alternat Med. 2022 Sep 17;2022:4731675. doi: 10.1155/2022/4731675 (PMC9509248; doi:10.1155/2022/4731675)
Supplement: Supplementary Materials — Supplementary Table 1: primer sequences used in qRT-PCR. [file 4731675.f1.docx]

**Supplementary table 1** Primer sequences used in qRT-PCR.

| RNA | Sequences (5’ to 3’) |
| --- | --- |
| TNF-α | F: 5’- GGTGCCTATGTCTCAGCCTCTT -3’ |
|  | R: 5’- GCCATAGAACTGATGAGAGGGAG -3’ |
| IFN-γ | F: 5’- CAGCAACAGCAAGGCGAAAAAGG -3’ |
|  | R: 5’- TTTCCGCTTCCTGAGGCTGGAT -3’ |
| IL-4 | F: 5’- CATCACTGCCACCCAGAAGACTG -3’ |
|  | R: 5’- ATGCCAGTGAGCTTCCCGTTCAG -3’ |
| Eotaxin | F: 5’ - TCCATCCCAACTTCCTGCTGCT -3’ |
|  | R: 5’- CTCTTTGCCCAACCTGGTCTTG -3’ |
| GAPDH | F: 5’- CATCACTGCCACCCAGAAGACTG -3’ |
|  | R: 5’- ATGCCAGTGAGCTTCCCGTTCAG -3’ |
